# Perceived Changes in Psychological Well-Being, Cognitive Domains, and Sleep Quality Among Jordanian Adults Observing Ramadan Intermittent Fasting: A Prospective Cohort Study

**DOI:** 10.3390/healthcare14193148

**Published:** 2026-09-22

**Authors:** Refat Ahmad Fawzi Alkurd, Narmeen Jamal Al-Awwad, Maysoun S. Qutob, Hani J. Hamad, Asma’a Albakri, Mohammed O. Ibrahim, Hadeel Soboh, Husam Khraiwesh, Fadwa Hammouh, Dana N. Abdelrahim, Hamid Aldeen Alhaj, MoezAlIslam E. Faris

**Affiliations:** 1 Department of Nutrition, Faculty of Pharmacy and Medical Sciences, University of Petra, Amman 11196, Jordan; 2 Science of Nutrition and Dietetics Program, College of Pharmacy, Al Ain University, Al Ain P.O. Box 64141, United Arab Emirates; narmeen.elawaad@aau.ac.ae; 3 Department of Clinical Nutrition and Dietetics, Faculty of Allied Medical Sciences, Applied Science Private University, Amman 11937, Jordan; m_alqutob@asu.edu.jo; 4 Department of Clinical Nutrition and Dietetics, Faculty of Allied Medical Sciences, Philadelphia University, Amman 19392, Jordan; hhamad@philadelphia.edu.jo; 5 Department of Nutrition and Food Technology, School of Agriculture, The University of Jordan, Amman 11942, Jordan; a.albakri@ju.edu.jo; 6 Department of Nutrition and Food Technology, Faculty of Agriculture, Mu’tah University, Karak 61710, Jordan; mohammedomar@mutah.edu.jo; 7 Department of Nutrition and Food Technology, Faculty of Agriculture, Jordan University of Science and Technology, Irbid 22110, Jordan; 8 Department of Nutrition and Food Processing, Faculty of Agricultural Technology, Al-Balqa Applied University, Salt 19117, Jordan; 9 Nutrition and Dietetics Department, Faculty of Health Sciences, American University of Madaba, Madaba 11821, Jordan; f.hammouh@aum.edu.jo; 10 Research Institute of Medical and Health Sciences (RIMHS), University of Sharjah, Sharjah P.O. Box 27272, United Arab Emirates; dabdel-rahim@sharjah.ac.ae; 11 Department of Family and Community Medicine and Behavioral Sciences, College of Medicine, University of Sharjah, Sharjah P.O. Box 27272, United Arab Emirates

**Keywords:** calorie restriction, time-restricted eating, mental health, psychology, nutritional neuroscience

## Abstract

**Background and Aims:** Ramadan intermittent fasting (RIF) involves significant shifts in sleep patterns and circadian rhythms that may affect neurological health. This study aimed to evaluate changes in neuropsychiatric and neurocognitive functions in relation to sleep quality among healthy adults observing RIF. **Methods:** We recruited healthy adults from various regions in Jordan for this prospective cohort study. We used validated self-report questionnaires to assess mental health and sleep parameters. Anxiety and depression were assessed using the GAD-7 and PHQ-9, respectively. We measured resilience using the 10-item Nicholson McBride Resilience Questionnaire (NMRQ), and the Neuro-QoL (cognitive functions domain) assessed memory and attention. We assessed sleep quality using the Pittsburgh Sleep Quality Index (PSQI). We administered all tools before (BR) and at the end of Ramadan (DR) to track changes in symptom severity and functional status. **Results:** A total of 723 healthy adults (mean age: 28.82 ± 12.04 years) completed both assessments. A paradoxical divergence emerged between mood and sleep parameters: mean scores for depression (PHQ-9: 8.65 to 7.70) and anxiety (GAD-7: 6.75 to 5.65; both *p* = 0.0001) improved significantly, with a higher proportion of participants reporting minimal symptoms during Ramadan. Conversely, overall sleep quality (PSQI) deteriorated markedly, with the percentage of participants classified as having “good sleep quality” dropping from 66.4% to 34.9% (*p* = 0.0001). While sleep latency and sleep disturbances improved slightly, subjective sleep quality and sleep duration worsened significantly (*p* ≤ 0.01). Additionally, Neuro-QoL cognitive scores decreased from 30.60 to 28.97 (*p* = 0.0001), whereas total resilience scores remained stable (*p* = 0.897). **Conclusions:** RIF is associated with significant improvements in depression and anxiety scores despite a concurrent deterioration in overall sleep quality and duration. While these findings suggest a favorable impact on subjective mental well-being, the observational design precludes establishing causal relationships. This paradoxical interplay between fasting-induced mood enhancements and sleep disruptions highlights the need for future prospective studies utilizing objective measurement tools.

## 1. Introduction

The global burden of mental health is a multifaceted challenge, with disorders contributing significantly to disability-adjusted life years and overall morbidity [1]. Approximately one in eight people worldwide lives with a mental disorder, with depression and anxiety affecting over 350 million individuals [2]. These conditions result in substantial social and economic sequelae, including reduced productivity and increased healthcare costs. Addressing this complexity requires integrated medical, social, and policy interventions to overcome disparities and limited access to services [3]. Dietary behaviors significantly influence the development and prognosis of mental health disorders. Emerging evidence suggests that intermittent fasting (IF) may benefit mental health by enhancing neuroplasticity and reducing the risk of neurodegenerative conditions [4,5]. These regimens may also impact mood regulation and stress resilience, with potential implications for depression and anxiety [6]. Mechanistically, IF may modulate neurotrophic factors, neurotransmitter systems, and inflammatory pathways [7]. Despite these promising initial findings, more rigorous research is required to establish the nuanced effects of specific IF regimens on mental well-being.

Sleep quality and psychological well-being are dynamically and bidirectionally linked [8,9]. Mounting research consistently links poor sleep hygiene to increased susceptibility to mood disturbances, including anxiety and depression [10]. These sleep disruptions alter emotional processing and mood regulation by affecting hormonal and neurotransmitter balances in the brain [11]. Conversely, while mental health problems often impair restorative sleep in a cyclical pattern, prioritizing good sleep hygiene has been shown to enhance emotional resilience [12].

Ramadan intermittent fasting (RIF) is a unique dietary and lifestyle transition involving abstinence from food and drink for 29–30 consecutive days. Unlike standard diurnal time-restricted eating (TRE) regimens, where the eating window typically aligns with daylight hours, it represents a unique chronobiological shift. RIF entails absolute abstinence from all food, liquids, and oral medications from dawn (*Fajr*) until sunset (*Maghrib*). Consequently, energy and fluid intake consolidate into the nocturnal period, accompanied by major adjustments in meal composition, delayed bedtimes, early awakenings for the pre-dawn meal (Suhoor), and late-night social and spiritual activities [13]. Beyond the 12–22 h daily fast, this shift from before Ramadan (BR) to during Ramadan (DR) entails drastic modifications in dietary intake [14], physical activity patterns [15], and sleep–wake cycles. These behavioral shifts induce significant circadian misalignment, altered core body temperature rhythms, and secondary partial sleep deprivation [16]. This circadian phase delay and sleep fragmentation can directly alter central neurotransmitter pathways, systemic inflammatory tone, and neuroendocrine signaling (e.g., cortisol and melatonin secretion), providing a distinct physiological backdrop for evaluating mental health and cognitive outcomes [16].

While earlier evidence regarding RIF and neurocognition was inconsistent, recent prospective data from a predominantly male cohort (84.2% male; n = 336) demonstrated that four weeks of dawn-to-dusk fasting are significantly associated with improved PHQ-9 depression and GAD-7 anxiety scores, as well as enhanced quality of life (QoL) [17]. Building on these observations, the present study extends this line of inquiry to a larger, more demographically diverse cohort. Recently, Elsahoryi et al. [18] investigated the impact of RIF in a small, single-center cohort of Jordanian university students (n = 77; mean age 21.2 ± 2.04 years). While their study provided valuable initial insights into changes in body composition and sleep quality, as assessed by the Pittsburgh Sleep Quality Index (PSQI) [18], it reported no statistically significant changes in overall mental health, as assessed by the General Depression Anxiety Stress Scales-21 (DASS-21). Still, important demographic and methodological gaps remain. Specifically, existing local literature is constrained by small, highly localized student samples and a lack of specialized neurocognitive evaluation. The present study addresses these gaps by evaluating a substantially larger, multi-regional cohort of Jordanian adults (N = 723; female-dominant) across a broader age spectrum (28.8 ± 12.0 years). Methodologically, we expand upon prior work by using a targeted, multi-domain psychometric battery-including the GAD-7 for anxiety, PHQ-9 for depression, the 10-item Nicholson McBride Resilience Questionnaire (NMRQ) for psychological coping capacity, and the Neuro-QoL scale, which specifically assesses cognitive functions (memory and attention)-alongside the PSQI. This approach provides a more robust evaluation of neuropsychiatric and neurocognitive shifts in relation to sleep quality during RIF among healthy people.

While primarily a spiritual practice, RIF is increasingly being studied for its potential impact on neuropsychiatric and neurocognitive functions. Existing literature often examines isolated metabolic or sleep indices, leaving a knowledge gap in how these circadian disruptions interact with psychological affect and cognitive perceptions. Given that current evidence remains complex and inconclusive, this study hypothesized that the systemic and behavioral shifts associated with RIF could mitigate factors contributing to mental health challenges despite sleep fragmentation. The primary objective was to evaluate the associations between RIF adherence and changes in subjective psychological well-being, perceptions of cognitive domains, resilience, and QoL. We also aimed to examine the complex interplay between these mental health shifts and changes in overall sleep quality during the transition from before Ramadan (BR) to the end of Ramadan (DR).

## 2. Methods and Participants

### 2.1. Research Design

The current work followed the Strengthening the Reporting of Observational Studies in Epidemiology (STROBE) guidelines [19,20]. This descriptive study employed a pre-post observational cohort design with a web-based survey, conducted in two stages: a week before Ramadan (17–25 March 2023) and during the last week of Ramadan (16–23 April 2023). The survey was an internet-based questionnaire administered via Google Forms, with closed-ended questions in Arabic, and was pretested for clarity. An inherent limitation of this observational prospective cohort design is the absence of a non-fasting control group. In a predominantly Muslim society such as Jordan, where observing RIF is a widespread socio-religious obligation, recruiting an eligible, socio-demographically matched non-fasting control group presents major ethical, practical, and logistical challenges. Consequently, while pre-to-post fasting comparisons allow for tracking intra-individual changes over time, this design constraint precludes definitive causal inferences regarding the observed neuropsychiatric and sleep quality outcomes.

To ensure a robust sample size, we collected baseline and follow-up data over approximately eight-day windows. The protocol ensured that each participant completed their follow-up assessment during the final week of Ramadan, after at least 3 consecutive weeks of fasting. The median interval between baseline (before Ramadan, BR) and follow-up (during Ramadan, DR) was 32 days (IQR: 30–34 days). This timing ensured that all participants had reached a significant threshold of cumulative physiological and behavioral exposure (approximately 22–28 consecutive days of fasting) by the follow-up assessment.

### 2.2. Study Population and Eligibility Criteria

To ensure a well-defined cohort, we applied explicit inclusion and exclusion criteria during baseline screening. Inclusion criteria were: (1) adults aged 18 to 65 years residing in Jordan; (2) intention to observe RIF for the full duration of the month; (3) ability to provide electronic informed consent and complete self-administered online questionnaires. Exclusion criteria were: (1) self-reported presence of chronic physical illnesses (e.g., cardiovascular disease, diabetes mellitus, severe gastrointestinal disorders, or chronic renal disease); (2) diagnosed neuropsychiatric, cognitive, or clinical sleep disorders; (3) current use of chronic medications that directly affect sleep, mood, or cognitive function (e.g., sedatives, hypnotics, antidepressants, or anxiolytics); (4) shift workers or individuals with irregular night-shift work schedules during the study period; (5) pregnancy or lactation. We embedded screening questions addressing these criteria at the beginning of the baseline survey to exclude ineligible respondents before administering the questionnaire.

### 2.3. Recruitment and Data Quality

Recruitment used digital convenience and snowball sampling via online social media platforms, introducing an inherent risk of selection bias. Reflecting typical user demographics of online survey sampling in the region, the final analytical sample was disproportionately composed of younger adults, females (~80%), and individuals with higher education levels. Consequently, these sampling constraints limit the direct generalizability of our findings to Jordan’s broader population (e.g., older adults, males, or those with lower levels of formal education), and this methodological limitation should be considered when interpreting the results.

We implemented several procedural safeguards to ensure data completeness and integrity across the two survey time points. First, we made all questionnaire items mandatory, preventing partial or incomplete submissions and eliminating item-level missing data. Second, the survey included embedded exclusion-criteria questions; participants who indicated ineligibility were automatically prevented from continuing or submitting the questionnaire, ensuring we captured only eligible responses at both time points. Third, we closed both the BR and DR questionnaires at the end of their respective designated survey periods. They were no longer accessible for submission thereafter, ensuring all responses were time-bound to the intended pre- and during-Ramadan windows. Of the 2199 participants who completed the BR questionnaire, 1083 (49.2%) subsequently completed the DR questionnaire. We linked participant records across the two time points using self-reported identifiers (email address, full name, and/or phone number), collected solely for matching purposes and accessed only by the study biostatistician to preserve confidentiality. Of the 1083 DR respondents, 745 matched to a corresponding BR record; 338 could not be matched because identifying information was missing or inconsistent across time points. We excluded an additional 22 records due to ambiguous matches, in which a shared identifier (e.g., a shared household phone number) corresponded to more than one possible baseline record, precluding confident pairing. This yielded a final analytical sample of 723 participants with uniquely and reliably matched pre- and during-Ramadan data (Figure 1).

### 2.4. Ethical Considerations and Informed Consent

The study followed the ethical principles of the Declaration of Helsinki. The Research Ethics Committee at the University of Sharjah, United Arab Emirates (UAE), reviewed and approved the protocol (Reference No: REC-23-03-13-01-F). Participants received a detailed information sheet at the start of the study, describing the research objectives, the 5–10 min time commitment required for the questionnaire, and the voluntary nature of participation. To ensure informed consent, participants were required to select ‘Agree’ on an electronic consent form before proceeding to the survey. The researchers strictly maintained participant anonymity and confidentiality throughout data collection and analysis, and informed all individuals of their right to withdraw from the study at any time without penalty.

### 2.5. Data Collection Tools and Scoring

Online, self-administered, validated questionnaires that assess anxiety, depression, resilience, and neuro QoL (Neuro-QoL), and subjectively assessed sleep quality at two points in time, before Ramadan (BR) and during Ramadan (DR). We distributed a web-based self-report questionnaire via social media platforms (e.g., WhatsApp, Facebook Messenger, Instagram, and Telegram) to a large audience in Jordan using snowball sampling.

The electronic survey was administered entirely in Arabic, the study population’s native language in Jordan. All psychometric instruments used in this investigation were either previously validated in Arabic or rigorously translated and adapted in accordance with established international guidelines. Specifically, we used validated Arabic versions of the Patient Health Questionnaire (PHQ-9) [21] and Generalized Anxiety Disorder scale (GAD-7) [22], with established internal consistency reported in prior validation studies (cohort-specific reliability values for all instruments are reported below, under Study instruments and psychometric evaluation). We evaluated subjective sleep parameters using the validated Arabic version of the Pittsburgh Sleep Quality Index (PSQI) [23]. We assessed cognitive domain perceptions using standardized Arabic translations of the World Health Organization Quality of Life Brief Version (WHOQOL-BREF) [24]. Because validated Arabic versions of the Quality of Life in Neurological Disorders (Neuro-QoL) scale and the NMRQ were not available in the literature, we formally translated them.

### 2.6. Pilot Testing Procedure

Before full study deployment, we conducted a formal pilot study with a convenience sample of 50 healthy adults residing in Jordan who met the study’s inclusion criteria. The primary objectives were to evaluate face and content validity, assess question clarity and linguistic appropriateness in Arabic, determine survey completion time (mean duration: 15–18 min), and test the digital platform’s technical functionality. For instruments requiring linguistic adaptation—specifically, the Neuro-QoL scale and the NMRQ, which lacked an established Arabic version—a standard forward–backward translation protocol was conducted by two independent bilingual experts in clinical nutrition and psychology, followed by verification by an expert panel. Pilot participants completed the full questionnaire battery and provided feedback on item ambiguity, formatting, and readability, which led to minor phrasing refinements. Across all administered instruments, we demonstrated strong internal reliability, with Cronbach’s alpha values ranging from 0.78 to 0.89 across domains. We excluded data from these 50 pilot participants from the final analytical dataset.

### 2.7. Sociodemographic, Anthropometric, and Lifestyle Factors

We collected baseline sociodemographic data, including sex, age, marital status, education, income, nationality, chronic diseases, and city of residence in Jordan. We collected information on smoking status, physical activity, supplement use, and anti-inflammatory medication use before and after Ramadan to assess changes. We recorded self-reported height only at baseline, and weight at baseline and during Ramadan. We calculated BMI from weight and height and categorized it according to WHO criteria [25].

### 2.8. Study Instruments and Psychometric Evaluation

To confirm the psychometric robustness of the instruments in our study population, we evaluated internal consistency using Cronbach’s alpha on the baseline dataset (N = 723). Internal consistency was good to excellent across all instruments. The PHQ-9 (α = 0.875), GAD-7 (α = 0.878), and NMRQ (α = 0.872) showed good-to-excellent reliability, while the Neuro-QoL showed excellent reliability (α = 0.956). The PSQI global score demonstrated acceptable reliability (α = 0.716), consistent with its multidimensional component structure.

### 2.9. Generalized Anxiety Disorder Questionnaire 7-Item (GAD-7)

The 7-item GAD questionnaire assesses anxiety over the past two weeks, covering feelings of nervousness, worry, difficulty, restlessness, irritability, and fear. Responses are scored from 0 (not at all) to 3 (nearly every day). Higher scores indicate greater anxiety severity, categorized as minimal (0–4), mild (5–9), moderate (10–14), and severe (15+) [26].

### 2.10. Patient Health Questionnaire 9-Item (PHQ-9)

The depression screening questionnaire has nine self-reported questions to assess the presence, severity, and monitoring of depression. Responses are scored from 0 (not at all) to 3 (nearly every day). Higher scores indicate more severe depression, categorized as minimal (0–4), mild (5–9), moderate (10–14), moderately severe (15–19), and severe (20–27) [21,27].

### 2.11. Resilience Questionnaire (NMRQ)

The resilience questionnaire is a shortened version of the Nicholson McBride Resilience Questionnaire (NMRQ) of 10 items on a 5-point Likert scale from 1 (strongly disagree) to 5 (strongly agree). Higher scores indicate greater resilience. Scores are categorized as developing (0–37), established (38–43), strong (44–48), and exceptional (49–60) [28].

### 2.12. Neuro-Quality of Life (QoL) Questionnaire (Cognitive Functions Domain) (Neuro-QoL)

The Neuro-QoL questionnaire assesses health-related QoL, focusing on cognitive functions like memory, attention, and daily task performance. The adult version includes an 8-item scale with two parts: the first four items measure event frequency (responses range from 1 = very often to 5 = not at all). The next four assess difficulty with daily tasks (responses range from 1 = cannot do to 5 = none). Scores are summed and converted to a T-score, where higher scores indicate better cognitive function. T-scores are categorized as cognitive impairment: severe (0–<30), moderate (30–40), mild (41–45), and normal (>45) [24,29].

### 2.13. Sleep Quality

Sleep quality was assessed using the Pittsburgh Sleep Quality Index (PSQI) [23], which comprises 19 items across seven components: subjective sleep quality, sleep latency, sleep duration, sleep efficiency, sleep disturbances, use of sleeping medication, and daytime dysfunction. Participants rated each item on a scale from 0 (good) to 3 (worst). Component scores ranged from 0 to 3 and were categorized as adequate (0–1) or inadequate (2–3). We calculated the total PSQI score (0–21) by summing the component scores, with higher scores indicating poorer sleep quality. Scores above 5 indicated poor sleep, while scores of 5 or below indicated good sleep quality [23,30]. We applied established psychometric cut-off thresholds to categorize subjective symptom severity across instruments (e.g., PHQ-9 and GAD-7 scores > 10 indicating moderate-to-severe symptoms; PSQI global score > 5 indicating poor sleep quality). These cut-offs are validated psychometric screening thresholds for self-reported symptoms, not diagnostic physiological criteria.

### 2.14. Data Analysis

We cleaned, scored, categorized, and encoded the data, then analyzed them using IBM Statistical Package for the Social Sciences (SPSS) Statistics (version 30; IBM Corp., Armonk, NY, USA) [31]. We summarized continuous variables as mean ± standard deviation (SD) and categorical variables as frequencies and percentages. We applied scale cutoffs as specified earlier. We assessed internal consistency reliability for each psychometric instrument using Cronbach’s alpha, with values ≥0.70 considered acceptable.

Following the reporting recommendations of Assel et al. (2019), we added statistical reporting measures to improve transparency and interpretability [32]. We compared pre- and post-Ramadan measures using paired *t*-tests for continuous variables and chi-square tests for categorical variables, as appropriate. In addition to *p*-values, we reported 95% confidence intervals (CIs) for all primary outcomes to improve interpretation of statistical precision. We calculated effect sizes (Cohen’s d) for key continuous outcomes to assess the magnitude and clinical relevance of observed changes. We used Spearman’s rank correlation coefficient to examine associations between study variables. All statistical tests were two-tailed, and a significance level of *p* < 0.05 was used. We followed the SAMPL guidelines throughout this project [33]. Paired comparisons of psychological and sleep outcomes between the pre- and during-Ramadan timepoints were conducted using repeated-measures analysis of covariance (ANCOVA), adjusting for physical activity frequency, dietary supplement use, and anti-inflammatory medication use as covariates; adjusted means, 95% confidence intervals, F-statistics, and partial eta-squared effect sizes are reported for each outcome; see Table S1.

## 3. Results

### 3.1. General Characteristics of the Study Participants

The study included 723 participants with a mean age of 28.82 ± 12.04 years. Most were female (80.4%), and about two-thirds were single, followed by married individuals. Most held a BSc (79.8%), and 11.8% held postgraduate degrees. Approximately 86% were Jordanians. Most resided in the Central region (88.8%), followed by the North (8.3%) and South (2.9%). The income distribution showed 35.7% in the low-income category, 28.4% in the lower-middle-income category, and 25.9% in the higher-middle-to-high-income category (Table 1).

### 3.2. Daily Lifestyle Behaviors and Anthropometric

The percentage of current smokers decreased significantly from 18.3% in BR to 16.6% in DR (*p* = 0.0001), while the percentage of non-smokers increased. Physical activity levels also changed, with a significant increase in the proportion of individuals who did not exercise during Ramadan (52.1% to 54.9%, *p* = 0.0001). The number of participants exercising 1–2 times, 3–5 times, or daily for DR declined. Additionally, more participants reported no physical activity (52.1% to 54.9%, *p* = 0.0001), with fewer engaging in over an hour of exercise (Table 2).

Table 2 also details changes in dietary supplement and anti-inflammatory medication use, with significant differences between BR and DR (*p* = 0.0001). Overall, notable shifts in smoking, physical activity, supplement, and medication habits suggest RIF’s potential impact on lifestyle. Body weight and BMI decreased significantly from baseline to the end of Ramadan, with favorable shifts in BMI classification (Table 2). Mean body weight significantly decreased from 68.13 ± 15.77 kg to 67.69 ± 15.40 kg (*p* = 0.002), corresponding to a reduction in mean BMI from 24.88 ± 4.60 kg/m^2^ to 24.73 ± 4.56 kg/m^2^ (*p* = 0.004). Categorical analysis revealed a slight increase in normal-weight participants (from 48.5% to 50.3%) with concurrent reductions across other categories (Table 2). Participants experienced a small but statistically significant reduction in body weight (mean difference = 0.45 kg, 95% CI: 0.16–0.74, Cohen’s d = 0.11, small effect, *p* = 0.002) and BMI (mean difference = 0.15 kg/m^2^, 95% CI: 0.05–0.25, Cohen’s d = 0.11, small effect, *p* = 0.004) during Ramadan compared with before Ramadan. These changes, although modest, indicate measurable improvements in anthropometric outcomes alongside significant shifts in physical activity and supplement use.

### 3.3. Neuropsychiatric and Neurocognitive Function

Table 3 provides a comprehensive overview of changes in neuropsychiatric and neurocognitive function parameters, such as depression, anxiety, resilience, and cognitive function (Neuro-QoL), reported by study participants before and during the Ramadan fasting month.

### 3.4. Depression (PHQ-9) Scale

Participants showed a statistically significant decrease in PHQ-9 depression score, from 8.65 ± 5.56 BR to 7.70 ± 5.47 DR (*p* = 0.0001). Results also revealed a significant shift, with an increase in individuals reporting none/minimal depression from 24.5% to 32.8% and a decrease in various depression severity categories. (Table 3)

### 3.5. Anxiety (GAD-7) Scale

Participants reported a statistically significant decrease in the anxiety (GAD-7) total score, declining from 6.75 ± 4.60 BR to 5.65 ± 4.59 DR (*p =* 0.0001). Categories demonstrate a notable increase in individuals experiencing minimal anxiety, from 37.6% BR to 46.9% DR, while showing noticeable reductions in the anxiety severity categories (mild to severe) (Table 3).

### 3.6. Resilience Scale

The overall resilience total score did not change significantly during Ramadan compared with the pre-fasting level. However, resilience categories increased in the developing and exceptional levels, while decreasing in the established and strong levels during Ramadan compared with pre-fasting levels (Table 3).

### 3.7. Quality of Life (QoL)

Neuro-QoL scores typically range from 0 to 100, with higher scores indicating better cognitive function and higher perceived QoL in mental abilities. Participants reported a statistically significant decrease in the Neuro-QoL total score (cognitive function domain), from 30.60 ± 7.11 BR to 28.97 ± 7.08 DR (*p* = 0.0001). The categories reflect a significant increase in individuals reporting severe cognitive impairment and a decrease in the other categories at the end of RIF (DR) compared with pre-fasting levels (BR) (Table 3).

Table 3 also showed that, during Ramadan, participants reported significant decreases in depression (PHQ-9; mean difference = 0.95, 95% CI: 0.58–1.32, Cohen’s d = 0.19, small effect, *p* < 0.001) and anxiety (GAD-7; mean difference = 1.11, 95% CI: 0.80–1.42, Cohen’s d = 0.26, small effect, *p* < 0.001). Cognitive function, measured via the Neuro-QoL domain, also showed a significant decline (i.e., worse perceived cognitive function) (mean difference = 1.27, 95% CI: 0.88–1.65, Cohen’s d = 0.24, small effect, *p* < 0.001), whereas resilience scores did not change significantly (mean difference = −0.04, 95% CI: −0.69–0.61, Cohen’s d = −0.005, negligible effect, *p* = 0.897).

### 3.8. Pittsburgh Sleep Quality Index (PSQI)

Subjective sleep quality (component 1) deteriorated, with scores increasing from 1.11 ± 0.79 to 1.23 ± 0.85 (*p* = 0.01), and the proportion reporting good sleep decreased from 72.8% to 66.4%. Sleep latency (component 2) improved, decreasing from 1.43 ± 1.04 to 1.16 ± 1.05 (*p* = 0.0001), and the proportion reporting adequate latency rose from 53.8% to 65.6%. Conversely, sleep duration (component 3) worsened, with scores increasing from 0.89 ± 0.86 to 1.26 ± 1.05 (*p* = 0.0001), and the proportion of adequate duration decreased from 80.5% to 65.0%. Sleep efficiency (component 4) increased slightly from 0.61 ± 1.02 to 0.62 ± 1.02 (*p* = 0.0001), with inadequate efficiency rising from 16.5% to 18.8%. Sleep disturbances (component 5) improved, with scores decreasing from 1.35 ± 0.58 to 1.23 ± 0.60 (*p* = 0.0001), and the percentage reporting no disturbances increased from 64.3% to 72.1%. Scores for sleep medication use (component 6) significantly decreased from 0.35 ± 0.77 to 0.27 ± 0.72 (*p* = 0.006), with adequate sleep without medication increasing from 88.9% to 92.0%. Daytime dysfunction (component 7) showed no significant change, with adequate function remaining stable.

Overall, the PSQI global score showed a decline in good sleep quality (<5) from 40.4% to 34.9%, while poor sleep quality (>5) rose from 59.6% to 65.1% after Ramadan. These results suggest that, despite improvements in sleep latency and disturbances, subjective sleep quality, duration, and overall sleep quality worsened, reflecting potential negative effects of Ramadan fasting on sleep patterns (Table 4). Sleep quality changes during Ramadan were mixed: subjective sleep quality (Component 1) showed a slight worsening (mean difference = −0.12, 95% CI: −0.18–−0.05, Cohen’s d = −0.13, small effect, *p* = 0.01), whereas sleep latency (Component 2; mean difference = 0.49, 95% CI: 0.36–0.62, Cohen’s d = 0.27, small effect, *p* < 0.001) and sleep duration (Component 3; mean difference = 0.57, 95% CI: 0.41–0.74, Cohen’s d = 0.26, small effect, *p* < 0.001) improved significantly. Use of sleep medications (Component 6) decreased modestly (mean difference = 0.08, 95% CI: 0.02–0.14, Cohen’s d = 0.10, small effect, *p* = 0.006), whereas overall global PSQI scores remained largely unchanged (mean difference = −0.04, 95% CI: −0.26–0.18, Cohen’s d = −0.01, negligible effect, *p* = 0.680) (Table 4).

### 3.9. Relationship Between Sleep and Cognitive Functions in the Context of RIF

Before Ramadan, a significant negative correlation (r = −0.113, *p* = 0.002) indicated that lower sleep quality was associated with decreased resilience. Positive correlations were observed between sleep quality and anxiety (0.450, *p* < 0.0001), depression (0.489, *p* < 0.0001), and cognitive function (0.360, *p* < 0.0001), suggesting that poorer sleep quality is related to higher anxiety and depression levels, but, surprisingly, higher cognitive function. During Ramadan, similar patterns persisted, with a negative correlation of −0.109 (*p* < 0.0001) between sleep quality and resilience, and positive correlations with anxiety (0.464), depression (0.484), and cognitive function (0.365), indicating that worse sleep quality during Ramadan was associated with increased anxiety and depression, and, unexpectedly, higher cognitive function (Table 5). These correlations offer insights into the relationships between sleep quality and other key variables, including resilience, anxiety, depression, and cognitive function, before and during Ramadan.

## 4. Discussion

This study provides a comprehensive exploration of the relationships among anxiety (GAD-7), depression (PHQ-9), resilience, QoL, and sleep quality (PSQI) in the context of RIF, filling a notable gap in lifestyle medicine research. Our findings—showing improved mental health and resilience despite declining sleep quality—align with studies across diverse populations, including those in Nigeria [34], Parkinson’s disease patients [35], and individuals with diabetes [36]. Similarly, reductions in stress and depression have been documented among fasting healthcare workers and during the COVID-19 pandemic, with RIF associated with enhanced life satisfaction and emotional well-being [37,38].

Resilience, the ability to adapt to adversity [39], was significantly enhanced in our cohort, consistent with previous research showing improved psychological hardiness and resilience among students during RIF [40]. Metabolic shifts during Ramadan, such as improved insulin sensitivity [41], may drive these improvements, as may modulation of neurotransmitters, including serotonin, dopamine, and endorphins, which stabilize mood and promote well-being [42,43]. Furthermore, RIF may foster neuroplasticity and cognitive resilience [44], complemented by the mindfulness, spiritual purpose, and communal support inherent to the month [45]. Reduced physiological stress, evidenced by lower cortisol levels [43], likely further alleviates anxiety.

### 4.1. Proposed Biological Mechanisms

The observed improvements in neurocognitive and neuropsychiatric functions during DR may reflect molecular adaptations induced by RIF’s unique metabolic state. Intermittent fasting has been shown to modulate the expression of key genes involved in mitochondrial biogenesis and antioxidant defense, including Nuclear Factor Erythroid 2-Related Factor 2 (*Nrf2*), Mitochondrial Transcription Factor A (*TFAM*), and Superoxide Dismutase 2 (*SOD2*) [46]. The upregulation of the Nrf2 pathway, in particular, is a critical mechanism for alleviating neuroinflammation and oxidative stress, both primary precursors of mental and cognitive decline. By enhancing neuroplasticity and maintaining mitochondrial integrity via these genetic regulators, the RIF model may provide a neuroprotective buffer that supports mental well-being and cognitive resilience, even amid altered sleep–wake cycles. Furthermore, the significant improvements in neurocognitive performance and neuropsychiatric scores from BR to DR may be driven by elevated levels of Brain-Derived Neurotrophic Factor (BDNF) [42,43]. This neurotrophic surge, likely triggered by the metabolic shift toward ketone body utilization during the 12–22 h fasting windows of RIF, is further supported at the molecular level by the upregulation of antioxidant and mitochondrial genes, including *Nrf2*, *SOD2*, and *TFAM* [46].

Beyond neurotrophic signaling, the improvements in neuropsychiatric well-being from BR to DR may reflect the potent anti-inflammatory effects of the RIF model. As established in our recent work on immune aging, intermittent fasting is a non-pharmacological intervention that mitigates immunosenescence and inflammaging, the chronic, low-grade systemic inflammation that predisposes individuals to neuroinflammation and frailty [47]. By suppressing pro-inflammatory cytokines and modulating the NLRP3 inflammasome [48], RIF likely reduces the inflammatory burden on the central nervous system. These pathways provide a robust biological explanation for the enhanced neurocognitive resilience and reduced anxiety and depression observed in our cohort, even amidst subjective sleep disruption.

Furthermore, the systemic shifts in the gut–brain axis during RIF likely contribute to the enhanced neuropsychiatric outcomes observed from BR to DR. As detailed in our recent review, the dietary and lifestyle changes during Ramadan can significantly affect gut health by modulating the gut microbiota and improving metabolic signaling [49]. This microbiomic remodeling, characterized by increased beneficial microbial metabolites such as short-chain fatty acids (SCFAs), works alongside the systemic reduction in ‘inflammaging’ and neuroinflammation [47]. Together with the upregulation of neurotrophic factors such as BDNF and antioxidant genetic pathways such as Nrf2, these synergistic mechanisms provide a comprehensive biological explanation for the neuro-resilience, reduced anxiety, and improved QoL observed in our Jordanian cohort, even amid subjective sleep disruption.

Our observation of a significant deterioration in global sleep quality (PSQI) aligns with recent findings among university students in Amman, in which RIF was associated with poorer subjective sleep quality, reduced sleep duration, and increased sleep disturbances. This reinforces the consensus that the nocturnal shifts inherent in RIF fundamentally alter restorative sleep patterns, a trend further reflected in the shared lifestyle shift toward reduced physical activity observed in both our cohort and other Jordanian settings. Interestingly, our findings contrast with this localized 2024 study of Jordanian students, which reported no significant changes in depression or anxiety during RIF. Our results, involving a significantly larger and more demographically diverse cohort (n = 723), revealed significant improvements in these parameters, suggesting that the neuroprotective or psychological benefits of RIF may be more detectable in larger populations or vary based on the specific psychometric tools employed, such as the GAD-7 and PHQ-9 used here versus the DASS-21 used in previous work [18].

### 4.2. Economic and Contextual Influences on Quality of Life

Overarching economic factors may strongly influence the observed reduction in QoL within the Jordanian context. During Ramadan and the subsequent celebration of Eid Al-Fitr, households face substantial financial strain from higher living costs, increased food expenditures, large family gatherings, and expectations of social hosting [50]. When superimposed on broader inflationary pressures, these acute financial demands can diminish the communal experience, generate baseline psychological stress, and potentially foster feelings of social exclusion [51], thereby directly modulating self-reported QoL and perceived performance in neurocognitive domains. Regarding sleep, while restorative sleep is essential for memory consolidation and executive function, the nocturnal social and religious rituals characteristic of Ramadan often disrupt sleep timing, architecture, and duration [52]. Consistent with prior literature [17,53,54], our cohort showed a marked deterioration in overall sleep quality. Large-scale epidemiological data indicate that during RIF, optimal sleep quality remains closely tied to lifestyle habits, with a preference for regular physical activity and plant-based diets. In contrast, behaviors like smoking and consumption of ultra-processed, ready-to-eat foods are associated with poorer sleep and mental health outcomes [52]. This paradoxical association between poorer subjective sleep and higher cognitive function is examined in detail below (see “Counterintuitive sleep and cognitive associations”). Notably, this is the first comprehensive assessment of these parameters in a healthy population, compared with prior clinical studies [55].

### 4.3. Counterintuitive Sleep and Cognitive Associations

An unexpected and counterintuitive finding of this study was the positive correlation between poorer subjective sleep quality (higher PSQI scores) and higher neurocognitive performance during Ramadan. While conventional sleep science typically indicates that sleep deprivation impairs cognitive function, our data suggest a more complex relationship within the unique context of RIF. We must acknowledge that this finding may be influenced by uncontrolled confounding variables, such as the cohort’s high educational level (which confers cognitive reserve) or the ‘motivational recruitment’ often observed during religious observance. Furthermore, this association may reflect measurement limitations inherent in self-report sleep scales, where a participant’s perceived sleep quality may not align with their actual physiological alertness. Given the speculative nature of these interpretations, view this correlation cautiously as an exploratory finding. It highlights the need for future studies that use objective sleep and cognitive metrics to determine whether the biological shifts associated with RIF truly provide a neuroprotective buffer against the typical cognitive costs of sleep disruption. The negative correlations with resilience and positive correlations with anxiety, depression, and cognitive function highlight potential associations between sleep quality and mental well-being in the study population.

Our observation of improved neuropsychiatric symptoms during DR aligns closely with the results of a recent investigation in the UAE, which also identified significant reductions in anxiety and depression scores during the final week of Ramadan [17]. Interestingly, while the UAE cohort showed improvements in subjective sleep quality (PSQI), our current Jordanian cohort reported a decline. This suggests that the neuropsychiatric benefits of RIF, specifically the reduction in anxiety and depression, may be a robust phenomenon that occurs independently of the specific direction of sleep quality changes.

To fully contextualize our findings, we must evaluate the observed changes in psychometric outcomes and sleep quality through the lens of circadian biology and the altered nocturnal dietary behaviors inherent to RIF. Unlike standard time-restricted eating protocols, in which food intake aligns with daylight hours, RIF shifts caloric, fluid, and nutrient intake entirely to the nocturnal window [13]. This radical shift in meal timing, often characterized by large, energy-dense meals at Iftar and late-night or pre-dawn Suhoor, profoundly affects peripheral clocks in metabolic organs, gastrointestinal motility, and postprandial thermogenesis during hours typically reserved for physiological rest. Concurrently, the necessity to awaken for Suhoor, coupled with late-night spiritual and social activities, induces a marked circadian phase delay, reduced total sleep duration, and altered sleep architecture (e.g., fragmentation of slow-wave and REM sleep) [16]. These combined chronobiological and dietary disruptions offer a clear physiological mechanism for the significant increase in subjective poor sleep quality (PSQI score > 5) and daytime fatigue reported by participants. Notably, despite these physiological and circadian perturbations, psychological metrics such as resilience (NMRQ) and perceived cognitive clarity (Neuro-QoL) remained preserved or showed positive self-perceived shifts, highlighting the powerful mitigating role of socio-religious engagement, spiritual mindfulness, and structured community support during the holy month.

### 4.4. Baseline Anticipatory Effects

A further consideration in interpreting our findings is the timing of the baseline assessment (BR), which occurred 1 week before the start of Ramadan. Some participants may already be making subtle shifts in sleep schedules, dietary habits, or social behaviors in anticipation of the holy month. While these “pre-Ramadan” adjustments may mean that the baseline does not represent a purely “neutral” non-fasting state, the primary physiological drivers of RIF, specifically, the prolonged daily fast and the significant nocturnal shift in caloric intake, were present only during the DR assessment. Therefore, the observed differences between BR and DR likely reflect the additional metabolic and circadian effects of the actual fasting month, beyond any minor anticipatory behavioral changes.

### 4.5. Limitations and Causal Interpretation

The limitations of this study span study design, measurement, and sample composition, and are grouped thematically below.

#### 4.5.1. Design Limitations

A primary methodological limitation is the absence of a non-fasting control group, which precludes directly attributing the observed neurocognitive and neuropsychiatric changes to fasting alone. However, establishing a concurrent non-fasting control group within the Jordanian population presents significant challenges, as RIF is a mandatory religious practice; those who do not fast typically consist only of individuals with specific medical exemptions or health excuses, which would introduce significant selection and health-status bias. Furthermore, while including non-fasting participants from different ethnic or racial groups was considered, such a comparison would likely yield inaccurate results due to confounding variables, including divergent genetic backgrounds and vastly different dietary, behavioral, and lifestyle norms. Consequently, the observed improvements should be interpreted as the result of the collective “Ramadan transition” -a multi-dimensional biocultural shift-rather than an isolated response to calorie or water restriction.

During Ramadan, several behavioral, social, and environmental factors shift simultaneously, including sleep–wake cycles, meal frequency, social engagement, and religious practices [16]. These co-occurring variables may confound the results, influencing sleep quality and cognitive function independently or synergistically. Consequently, the observed improvements should be interpreted as the result of the collective “Ramadan transition”, a multi-dimensional biocultural shift, rather than an isolated response to calorie or water restriction. Future studies using a crossover design in non-Muslim-majority settings or employing high-resolution ecological momentary assessment (EMA) could help further disentangle these specific behavioral drivers.

A further limitation of the study design is the single follow-up point during the final week of Ramadan (DR). This approach focuses on the cumulative and stabilized effects of the month-long fast. Still, it does not capture the acute phase of physiological and behavioral adaptation that typically occurs during the first week of Ramadan. Early-month transitions often involve transient symptoms, including increased irritability, headaches, and acute disruption of the sleep–wake cycle, as the body adjusts to new feeding and sleeping windows. Consequently, our findings reflect participants’ sustained neuropsychiatric and neurocognitive state during the latter stage of the month, rather than the dynamic shifts of the initial adaptation period.

Beyond the fasting protocol itself, several behavioral, social, and environmental factors shift simultaneously during Ramadan—including sleep–wake cycles, meal frequency and composition, physical activity, dietary supplement intake, anti-inflammatory medication use, and social and religious engagement [16]—and may act as confounders, independently or synergistically influencing sleep quality, mood, and cognitive function. For instance, reduced moderate-to-vigorous physical activity during fasting hours may independently affect mood indices and sleep architecture, while changes in supplement or medication compliance could alter systemic inflammatory tone and perceived energy levels. Accounting for these co-occurring lifestyle shifts helps isolate RIF’s distinct contribution while recognizing the holistic, multifactorial nature of behavioral change during the holy month. Future studies using a crossover design in non-Muslim-majority settings or high-resolution EMA could help disentangle these specific behavioral drivers.

#### 4.5.2. Measurement Limitations

This study relied exclusively on self-administered psychometric questionnaires (e.g., PSQI, Neuro-QoL, PHQ-9, GAD-7, and NMRQ), which are inherently susceptible to recall, response, and social desirability biases. While these validated tools effectively capture subjective perceptions of sleep quality and neuropsychiatric well-being, self-reported scores may not perfectly correlate with underlying physiological markers or clinical neurocognitive performance. This distinction is particularly relevant for the cognitive domain: cognitive function was evaluated solely via self-reported subscales (Neuro-QoL), which capture perceived cognitive clarity, focus, and subjective daily functioning rather than objective gains in executive function, working memory, or processing speed. In the context of secondary sleep deprivation and altered sleep–wake schedules, such positive self-perceptions may instead be mediated by heightened spiritual mindfulness, structured daily routines, strong social support, and a sense of collective accomplishment during the holy month. These are socio-cultural and psychological drivers that can cushion subjective well-being despite physiological fatigue. The absence of direct objective testing, such as computerized psychomotor vigilance, reaction time, or executive function tasks, or objective sleep measures such as actigraphy or polysomnography (PSG) precludes definitive conclusions regarding actual neurocognitive or sleep changes during Ramadan fasting. Caution is therefore warranted to avoid overinterpreting self-reported instrument scores as evidence of objective enhancement, and future investigations should incorporate objective performance and sleep-monitoring batteries alongside self-report metrics.

#### 4.5.3. Sample Limitations

The study used convenience and snowball sampling through social media platforms, introducing selection bias and limiting the representativeness of the findings. The resulting cohort is heavily skewed toward younger, highly educated females. This demographic may be more cognitively engaged or have different baseline neuropsychiatric profiles than the general adult population observing Ramadan. Consequently, our findings may not be fully generalizable to older adults, individuals with lower educational attainment, or those with pre-existing metabolic or psychiatric conditions. In this demographic, the observed improvements in neurocognitive and neuropsychiatric scores might reflect a greater capacity for behavioral adaptation to the circadian and dietary shifts associated with RIF. Future research should use stratified random sampling to ensure broader demographic representation and determine whether these positive associations persist across diverse age and socioeconomic strata. Although our convenience sample was predominantly female, the reproducibility of these neuropsychiatric improvements in a cohort that was 84.2% male [17] suggests that the observed associations between RIF and improved mental well-being are likely generalizable across sexes within the Middle Eastern context. Furthermore, the unique spiritual, communal, and socio-cultural dimensions inherent to RIF represent a distinct behavioral context that non-religious or experimental IF protocols cannot fully replicate. While this limits the direct translation of our findings to non-religious IF regimens, it significantly strengthens the external validity and generalizability of our results to Muslim individuals observing RIF globally across different geographic and cultural settings.

A further limitation is that we could not compare baseline characteristics between completers and non-completers due to data availability constraints; therefore, we cannot rule out the possibility that attrition introduced selection bias into the final analytical sample.

#### 4.5.4. Interpretive Limitations and Future Directions

Lastly, when interpreting the study findings, we must clearly distinguish statistical significance from clinical meaningfulness. Due to the statistical power afforded by our large analytical sample (N = 723), minor psychometric shifts were highly statistically significant (*p* = 0.0001). However, the calculated effect sizes were small (Cohen’s d = 0.19 for depression [PHQ-9] and *d* = 0.26 for anxiety [GAD-7]). For example, the observed 0.95-point decrease in PHQ-9 mean score (from 8.65 ± 5.56 to 7.70 ± 5.47) falls below established minimal clinically important difference (MCID) thresholds for clinical intervention in psychiatric populations. In a generally healthy adult population, these changes reflect subtle, positive fluctuations in subjective well-being and psychological affect during Ramadan rather than major therapeutic or clinical remissions. Acknowledging this distinction prevents overinterpreting modest population-level trends as dramatic clinical benefits. Another limitation is that we could not compare baseline characteristics between completers and non-completers because of data availability constraints; therefore, we cannot rule out the possibility that attrition introduced selection bias into the final analytical sample.

To address current methodological constraints—namely, the lack of a non-fasting control group, reliance on retrospective self-report questionnaires, selection bias from online convenience sampling, and low temporal resolution—future studies should adopt more rigorous design strategies. Specifically, employing randomized crossover trial designs—comparing fasting and non-fasting phases within the same individuals across non-religious periods or in non-observing control cohorts—would help isolate the specific metabolic effects of time-restricted feeding from socio-cultural confounders. Additionally, implementing EMA via mobile devices would enable researchers to capture real-time, micro-level fluctuations in mood, subjective sleepiness, cognitive fatigue, and dietary intake in participants’ natural environments throughout the holy month, thereby significantly minimizing recall bias and providing high temporal resolution.

## 5. Conclusions

This study demonstrates that observing RIF is associated with significant improvements in depression and anxiety symptoms, despite a simultaneous and sharp decline in objective sleep quality and duration. While the improvements in neuropsychiatric parameters are promising, the observational nature of this study precludes establishing a direct causal link between fasting and mental health outcomes. Furthermore, the decline in sleep quality and its correlation with mental health highlights the complex, multifaceted impact of RIF on well-being. These findings underscore the need for future longitudinal research using objective clinical measures to explore these relationships further and better understand the underlying mechanisms.

## Figures and Tables

**Figure 1:**
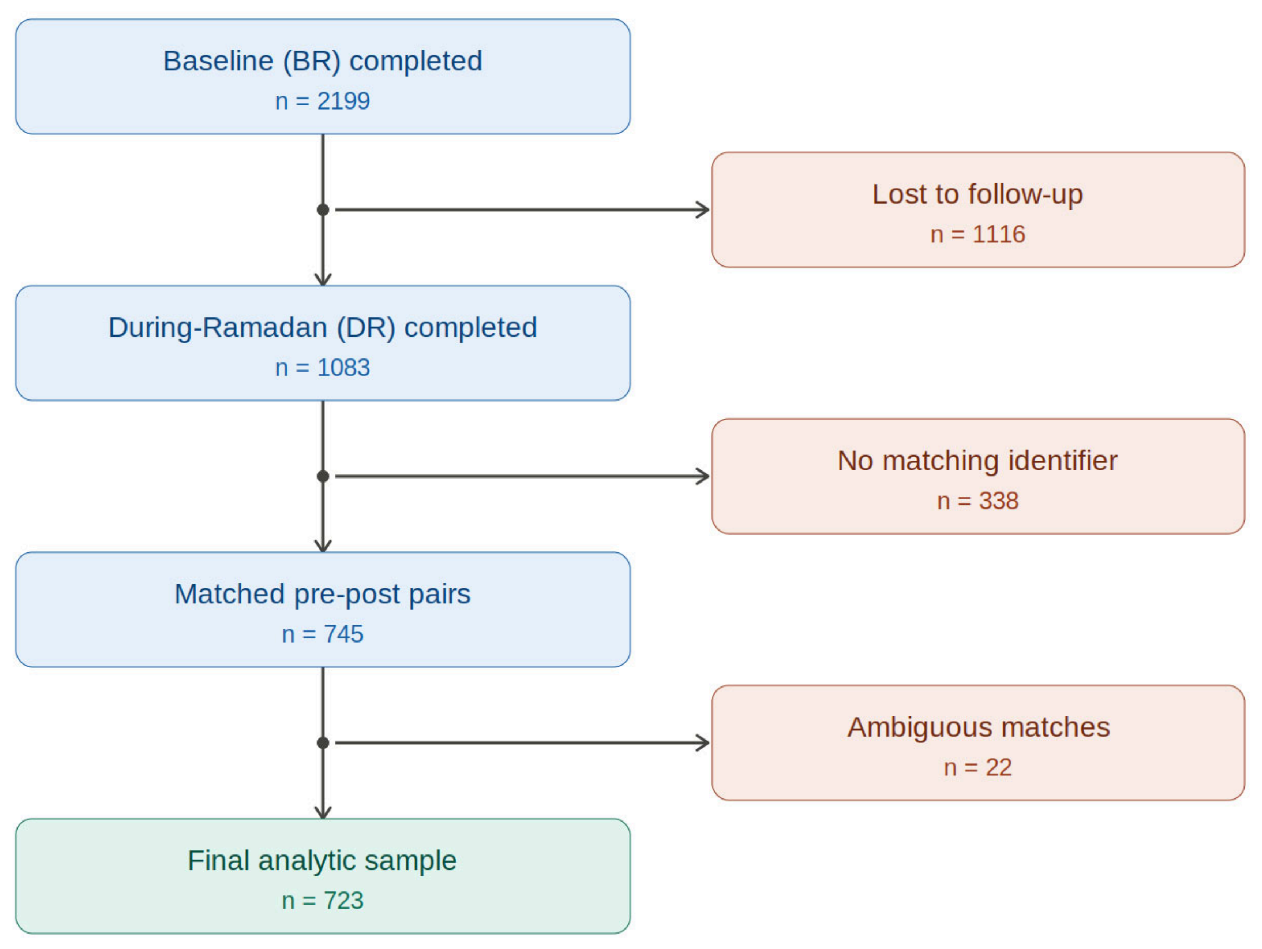
Flow diagram of participant enrollment, follow-up, matching, and exclusion across the pre- and during-Ramadan assessment periods.

**Table 1:** The basic health and sociodemographic characteristics of the study participants (n = 723).

Variables		n	%
Sex	Female	581	80.4
	Male	142	19.6
Marital status	Single	472	65.3
	Married	228	31.5
	Widowed	5	0.7
	Divorced	18	2.5
Education	Primary and secondary	61	8.4
	University BSc	577	79.8
	MSc and PhD	85	11.8
Nationality	Jordanian	620	85.8
	Non-Jordanian	103	14.2
Residence	Central	642	88.8
	North	60	8.3
	South	21	2.9
Income * (JD/month)	<240 (Low)	258	35.7
	240–846 (Lower-Middle)	278	28.4
	>846 (Higher-Middle to High)	187	25.9
Diagnosed with a chronic disease	Yes	145	20.1
	No	578	79.9

* Jordan Dinar (JD).

**Table 2:** Changes in anthropometric characteristics, lifestyle behaviors, and medication/supplement use of the study participants before and during the Ramadan fasting month (n = 723) [n (%)].

Variable		BR	DR	Mean Difference (95% CI)	Cohen’s d	*p*-Value
Smoking (currently)	Smoker	132 (18.3%)	120 (16.6%)	-	-	0.0001 *
	Non-smoker	591 (81.7%)	603 (83.4%)	-	-	
Physical activity (PA) frequency	No PA	377 (52.1%)	397 (54.9%)	-	-	0.0001 *
	1–2 times a week	229 (31.7%)	221 (30.6%)	-	-	
	3–5 times a week	88 (12.2%)	82 (11.3%)	-	-	
	Daily	29 (4.0%)	23 (3.2%)	-	-	
Physical activity (PA) duration (hours/day)	No PA	377 (52.1%)	397 (54.9%)	-	-	0.0001 *
	Less than half an hour	110 (15.2%)	123 (17.0%)	-	-	
	Half hour to one hour	165 (22.8%)	155 (21.4%)	-	-	
	More than 1 h	71 (9.9%)	48 (6.7%)	-	-	
Use dietary supplements	Yes	304 (42.0%)	278 (38.5%)	-	-	0.0001 *
	No	419 (58.0%)	445 (61.5%)	-	-	
Use of anti-inflammatory medications	Yes	73 (10.1%)	72 (10.0%)	-	-	0.0001 *
	No	650 (89.9%)	651 (90.0%)	-	-	
Body weight (kg)		68.13 ± 15.77	67.69 ± 15.40	0.447 (0.158–0.735)	0.113	0.002 *
BMI (kg/m^2^)		24.88 ± 4.60	24.73 ± 4.56	0.149 (0.048–0.251)	0.108	0.004 *
BMI category (kg/m^2^)	Underweight	49 (6.8%)	48 (6.6%)	-	-	0.0001 *
	Normal weight	351 (48.5%)	364 (50.3%)	-	-	
	Overweight	227 (31.4%)	219 (30.3%)	-	-	
	Obese	96 (13.3%)	92 (12.7%)	-	-	
PSQI Global Score	Good sleep quality (≤5)	292 (40.4%)	252 (34.9%)	-	-	0.0001 *
	Poor sleep quality (>5)	431 (59.6%)	471 (65.1%)	-	-	

Mean differences are presented as (DR–BR) with 95% confidence intervals (CIs). Effect sizes were calculated using Cohen’s d for paired samples. *p*-values were obtained using paired *t*-tests (continuous variables) and chi-square tests (categorical variables). * Statistical significance at *p* < 0.05.

**Table 3:** Changes in depression, anxiety, resilience, and neuro-quality of life (QoL, cognitive function domain) scales reported by the study participants before and during the Ramadan fasting month (n = 723).

Variable		BR	DR	Mean Difference (95% CI)	Cohen’s d	*p*-Value
PHQ-9 total score		8.65 ± 5.56	7.70 ± 5.47	0.949 (0.575–1.323)	0.185	0.0001 *
PHQ-9 categories	None/minimal depression (0–4)	177 (24.5%)	237 (32.8%)	-	-	0.0001 *
	Mild depression (5–9)	297 (41.1%)	278 (38.5%)	-	-	
	Moderate depression (10–14)	139 (19.2%)	123 (17.0%)	-	-	
	Moderately severe depression (15–19)	71 (9.8%)	58 (8.0%)	-	-	
	Severe depression (20–27)	39 (5.4%)	27 (3.7%)	-	-	
GAD-7 total score		6.75 ± 4.60	5.65 ± 4.59	1.105 (0.796–1.415)	0.261	0.0001 *
GAD-7 categories	Minimal anxiety (0–4)	272 (37.6%)	339 (46.9%)	-	-	0.0001 *
	Mild anxiety (5–9)	281 (38.9%)	262 (36.2%)	-	-	
	Moderate anxiety (10–14)	119 (16.5%)	81 (11.2%)	-	-	
	Severe anxiety (more than 15)	51 (7.1%)	41 (5.7%)	-	-	
Resilience total score		39.44 ± 8.03	39.48 ± 8.64	−0.043 (−0.694–0.609)	−0.005	0.897
Resilience categories	Developing level (0–37)	251 (34.7%)	261 (36.1%)	-	-	0.0001 *
	Established level (38–43)	242 (33.5%)	222 (30.7%)	-	-	
	Strong level (44–48)	174 (24.1%)	170 (23.5%)	-	-	
	Exceptional level (49–60)	56 (7.7%)	70 (9.7%)	-	-	
Neuro-QoL total score (Cognitive function domain)		30.60 ± 7.11	28.97 ± 7.08	1.267 (0.884–1.650)	0.242	0.0001 *
Neuro-QoL categories	Severe (0–30)	358 (49.5%)	420 (58.1%)	-	-	0.0001 *
	Moderate (30.001–40)	311 (43.0%)	267 (36.9%)	-	-	
	Mild (40.001–45)	38 (5.3%)	26 (3.6%)	-	-	
	Within normal limits (45.001–80)	16 (2.2%)	10 (1.4%)	-	-	

Mean differences are presented as (DR–BR) with 95% confidence intervals (CIs). Effect sizes were calculated using Cohen’s d for paired samples. *p*-values were obtained using paired *t*-tests (continuous variables) and chi-square tests (categorical variables). * Statistical significance at *p* < 0.05.

**Table 4:** Changes in Pittsburgh Sleep Quality Index (PSQI) and its components before and during the Ramadan fasting month for the study participants (n = 723).

		BR	DR	Mean Difference (95% CI)	Cohen’s d	*p*-Value
PSQI Component 1: Subjective Sleep Quality		1.11 ± 0.79	1.23 ± 0.85	−0.116 (−0.183–−0.050)	−0.127	0.01 *
	Adequate (0–1)	526 (72.8%)	480 (66.4%)	-	-	0.0001 *
	Inadequate (2–3)	197 (27.2%)	243 (33.6%)	-	-	
PSQI Component 2: Sleep Latency		1.43 ± 1.04	1.16 ± 1.05	0.487 (0.355–0.619)	0.270	0.0001 *
	Adequate (0–1)	389 (53.8%)	474 (65.6%)	-	-	0.0001 *
	Inadequate (2–3)	334 (46.2%)	249 (34.4%)	-	-	
PSQI Component 3: Sleep Duration		0.89 ± 0.86	1.26 ± 1.05	0.573 (0.411–0.736)	0.258	0.0001 *
	Adequate (0–1)	582 (80.5%)	470 (65.0%)	-	-	0.0001 *
	Inadequate (2–3)	141 (19.5%)	253 (35.0%)	-	-	
PSQI Component 4: Sleep efficiency		0.61 ± 1.02	0.62 ± 1.02	−36.22 (−62.11–−10.33)	−0.103	0.0001 *
	Adequate (0–1)	604 (83.5%)	587 (81.2%)	-	-	0.0001 *
	Inadequate (2–3)	119 (16.5%)	136 (18.8%)	-	-	
PSQI Component 5: Sleep disturbances		1.35 ± 0.58	1.23 ± 0.60	0.985 (0.663–1.306)	0.224	0.0001 *
	Adequate (0–1)	465 (64.3%)	521 (72.1%)	-	-	0.0001 *
	Inadequate (2–3)	258 (35.7%)	202 (27.9%)	-	-	
PSQI Component 6: Use of sleep medications		0.35 ± 0.77	0.27 ± 0.72	0.082 (0.023–0.140)	0.102	0.006 *
	Adequate (0–1)	643 (88.9%)	665 (92.0%)	-	-	0.0001 *
	Inadequate (2–3)	80 (11.1%)	58 (8.0%)	-	-	
PSQI Component 7: Daytime dysfunction		1.10 ± 0.80	1.12 ± 0.87	−0.059 (−0.177–0.058)	−0.037	0.538
	Adequate (0–1)	512 (70.8%)	505 (69.8%)	-	-	0.0001 *
	Inadequate (2–3)	211 (29.2%)	218 (30.2%)	-	-	
PSQI Global Score		6.85 ± 3.32	6.90 ± 3.09	−0.041 (−0.261–0.178)	−0.014	0.680
	Good sleep quality (≤5)	292 (40.4%)	Good sleep quality (≤5)	-	-	0.0001 *
	Poor sleep quality (>5)	431 (59.6%)	Poor sleep quality (>5)	-	-	

Mean differences are presented as (DR–BR) with 95% confidence intervals (CIs). Effect sizes were calculated using Cohen’s d for paired samples. *p*-values were obtained using paired *t*-tests (continuous variables) and chi-square tests (categorical variables). * Statistical significance at *p* < 0.05.

**Table 5:** Correlations between neuropsychiatric and neurocognitive function variables and subjectively assessed sleep quality (PSQI) before and during Ramadan for study participants (n = 723).

Time Point	Neuropsychiatric and Neurocognitive Function	PSQI Global Score
BR	Anxiety (GAD-7)	0.450 (0.0001) **
	Depression (PHQ-9)	0.489 (0.0001) **
	Resilience	−0.113 (0.002) **
	Neuro-QoL	0.360 (0.0001) **
DR	Anxiety (GAD-7)	0.464 (0.0001) **
	Depression (PHQ-9)	0.484 (0.0001) **
	Resilience	−0.109 (0.0001) **
	Neuro-QoL	0.365 (0.0001) **

** Statistical significance at *p* < 0.001.

## Data Availability

The data that support the findings of this study are available from the corresponding author, M.E.F., upon reasonable request. The data are not publicly available due to privacy and ethical restrictions, as they contain information that could compromise the privacy of research participants in accordance with the IRB protocols of the University of Sharjah and participating institutions.

## Supplementary Materials

The following supporting information can be downloaded at: https://www.mdpi.com/article/10.3390/healthcare14193148/s1, Table S1. Repeated-measures ANCOVA of psychological and sleep outcomes before and during Ramadan, adjusted for physical activity, changes in supplement use, and anti-inflammatory medication use.
