# Supplementary material for: Perceived Changes in Psychological Well-Being, Cognitive Domains, and Sleep Quality Among Jordanian Adults Observing Ramadan Intermittent Fasting: A Prospective Cohort Study
Source: Healthcare (Basel). 2026 Sep 22;14(19):3148. doi: 10.3390/healthcare14193148 (PMC13665458; doi:10.3390/healthcare14193148)
Supplement: Supplementary file 1 [file healthcare-14-03148-s001.zip › healthcare-4435112-supplementary.pdf]

Supplementary Table S1. Repeated-measures ANCOVA of psychological and sleep outcomes before and during Ramadan, adjusted for physical activity, changes in supplement use, and anti-inflammatory medication use

**Outcome**	**Adjusted Mean BR (95% CI)**	**Adjusted Mean DR (95% CI)**	**F**	**df**	**p-value**	**Partial η²**
PHQ-9	8.50 (7.87–9.12)	7.22 (6.61–7.84)	3.618	1, 717	**0.051**	0.005
GAD-7	6.65 (6.13–7.17)	5.32 (4.80–5.83)	6.16	1, 717	**0.013**	0.009
QOL	30.23 (29.43–31.03)	28.50 (27.70–29.30)	6.103	1, 717	**0.014**	0.008
NMRQ	40.23 (39.33–41.13)	39.73 (38.77–40.70)	2.964	1, 717	0.086	0.004
PSQI	6.81 (6.44–7.18)	6.77 (6.42–7.12)	1.477	1, 717	**0.025**	0.002

Note. BR = before Ramadan; DR = during Ramadan; CI = confidence interval; GAD-7 = Generalized Anxiety Disorder-7; PHQ-9 = Patient Health Questionnaire-9; QOL = Neuro-QoL Cognitive Domain; NMRQ = Nicholson McBride Resilience Questionnaire; PSQI = Pittsburgh Sleep Quality Index. Adjusted means were derived from repeated-measures ANCOVA with Time (BR vs. DR) as the within-subject factor, controlling for physical activity frequency (categorical), dietary supplement use, and anti-inflammatory medication use at baseline. $p < .05$ was considered statistically significant.
